# Herbal Infusions as a Valuable Functional Food

**DOI:** 10.3390/nu13114051

**Published:** 2021-11-12

**Authors:** Elżbieta Studzińska-Sroka, Agnieszka Galanty, Anna Gościniak, Mateusz Wieczorek, Magdalena Kłaput, Marlena Dudek-Makuch, Judyta Cielecka-Piontek

**Affiliations:** 1 Department of Pharmacognosy, Poznan University of Medical Sciences, Swiecickiego 4, 60-781 Poznań, Poland; annagos97@gmail.com (A.G.); mateuszwieczorek23@gmail.com (M.W.); dudum@poczta.onet.pl (M.D.-M.); jpiontek@ump.edu.pl (J.C.-P.); 2 Department of Pharmacognosy, Faculty of Pharmacy, Jagiellonian University Medical College, Medyczna 9, 30-688 Kraków, Poland; agnieszka.galanty@uj.edu.pl; 3 Department of Pediatric Gastroenterology and Metabolic Diseases, Poznan University of Medical Sciences, 27/33 Szpitalna Str., 60-572 Poznań, Poland; magdalena.klaput@ump.edu.pl

**Keywords:** antidiabetic activity, polyphenols, antioxidant activity, inhibition of α-glucosidase, inhibition of α-amylase, inhibition of collagenase

## Abstract

Herbal infusions are an underestimated and easy to intake a source of biologically active natural compounds (polyphenols), which, in the dissolved form, are more easily absorbed. Therefore, this study aimed to assess the potential of herbal infusions as a functional food to reduce postprandial hyperglycemia (inhibition of α-amylase and α-glucosidase) and to reduce the effects of increased blood glucose level (antioxidant effect-DPPH, CUPRAC, and Fe^2+^ chelating assays, as well as anti-inflammatory activity-inhibition of collagenase). We showed that polyphenols are present in the examined aqueous herbal infusions (including chlorogenic and gallic acids). Subsequently, our research has shown that herbal infusions containing cinnamon bark, mulberry leaves, and blackberry fruits most strongly inhibit glucose release from complex carbohydrates, and that all herbal infusions can, to different degrees, reduce the effects of elevated blood sugar. In conclusion, infusions prepared from herbal blends could be recommended to prevent type II diabetes.

## 1. Introduction

Diabetes is a metabolic disease characterized by a chronic condition of hyperglycemia. According to the World Health Organization (WHO), it already affects 422 million adults worldwide [1]. As a result of diabetes development, the process of glycation of proteins, lipids, and nucleotides increases due to the persistence of high blood glucose levels. The reactive dicarbonyl molecules formed as a result of this glycation react with the amino groups of proteins, leading to the synthesis of advanced irreversible glycation products (AGEs). AGEs, by connecting to a specific receptor for advanced glycation end-products receptor found on the surface of e.g., lymphocytes, cardiomyocytes, or neurons of the central and peripheral nervous system, activate appropriate transcription factors, and induce the synthesis of reactive oxygen species. This intensifies the oxidation of glucose (glycoxidation) and lipids (lipoxidation), leading to the development and management of diabetes complications such as retinopathy, nephropathy, and diabetic neuropathy [2].

The development of diabetic complications also results from the insufficient effectiveness of pharmacotherapy of synthetic drugs [3]. Given the aging population, the trend of developing diabetes and the limited efficacy of synthetic drug therapy will be increasing. The strategy of combating type II diabetes involves also the change in the pharmacotherapy, from the use of old drug groups (e.g., sulfonylurea derivatives) that act directly in pancreatic cell membranes, and often produce side effects [4,5], to new therapeutic groups (e.g., dipeptidyl peptidase-4 inhibitors), based on the mechanisms of action in the intestine [6]. The compounds inhibiting α-glucosidase and α-amylase also have their mechanism in the intestine. Glucosidases are responsible for the reduction of dietary carbohydrates into simple sugars that are quickly absorbed by the small intestine [7]. The α-glucosidase and α-amylase inhibitors can reduce hyperglycemia, and decreased the side effects of hyperglycemic drugs.

Diet is an important factor in proper carbohydrate metabolism. Dietary ingredients not only affect the reduction of postprandial glucose, but also may even regulate blood sugar [8]. The most important mechanisms of natural compounds for lowering sugar into the blood include: inhibiting their absorption, increasing their metabolism by stimulating insulin secretion and degradation in peripheral cells, and accelerating sugar excretion by inducing increased renal diuresis [9,10]. Raw plant materials are often used to support type II diabetes as herbal drugs. According to the guidelines of the Committee on Herbal Medicinal Products, raw plant materials can be registered as herbal drugs as a result of their well-established use and traditional use procedure [11]. Therefore, the preventive or curative effects of herbal raw materials can be a significant support in combating diabetes, together with a proper diet or using appropriate functional food. The compounds most responsible for the antidiabetic activity of plant materials include: flavonoids, anthocyanins, phenolic acids, some polysaccharides such as inulin which are not degradable to monosaccharides, alkaloids (deoxynojirimycin, found in mulberry leaves), as well as tannins or ingredients of essential oils, including cinnamaldehyde, found in a large amount in the cinnamon bark [12].

Herbal infusions are an easy-to-apply form of the herbs, which is an important argument, especially for senior patients with swallowing problems. Plant infusions also provide a very good distribution of active compounds in the intestine, which results in their effectiveness. The significant availability of a variety of herbal blends (HBs) for infusions preparations in the markets of each country is also important. In the view of the “silent epidemic” of diabetes II, hypoglycemic infusions can be an important element in combating the development of this disease.

In this way, our study aimed to investigate the antidiabetic potential of HBs infusions dedicated as supportive treatment of diabetes. 

## 2. Materials and Methods

### 2.1. Herbal Tea Blends 

Seven HBs, often recommended to patients or people at risk of developing diabetes II, were included in the study. The tested HBs were all produced by Polish herb packaging companies, and were purchased in pharmacies and herbal stores in Poznan, Poland. The detailed compositions of the blends are presented in Table 1.

### 2.2. Chemical Reagents 

Ethanol, Folin–Ciocalteu reagent, (Merck, Darmstadt, Germany), 3,5-dinitrosalicylic acid, 2,2-diphenyl-1-picrylhydrazyl (DPPH), 4-nitrophenyl α-D-glucopyranoside (PNPG), α-glucosidase, α-amylase, acetonitrile, collagenase from *Clostridium histolyticum*, formic acid, iron (II) chloride tetrahydrate, ferrozine, N-[3-(2-Furyl)acryloyl]-Leu-Gly-Pro-Ala (FALGPA), neocuproine, tricine, (Sigma-Aldrich, St. Louis, MO USA); aluminum chloride anhydrous, ammonium acetate, calcium chloride, copper (II) chloride dihydrate, disodium hydrogen phosphate dodecahydrate, methanol, sodium carbonate anhydrous, sodium dihydrogen phosphate dehydrate, sodium chloride, sodium hydroxide, and sodium phosphate monobasic (Avantor Performance Materials Poland S.A., Gliwice, Poland), were used. Standards: gallic acid and acarbose, ethylenediaminetetraacetic acid (EDTA) (Sigma-Aldrich, St. Louis, MO USA), chlorogenic acid, protocatechuic acid, quercetin, and rutin (Carl Roth GmbH + Co. KG, Karlsruhe, Germany) were used.

### 2.3. Extracts Preparations

The infusions were prepared by pouring a 2-g fix sachet with 200 mL of boiling distilled water. For different weight sachets, the amount of water was proportional. Herbs were infused under a cover for 15 min. The resulting infusion was concentrated using a vacuum evaporator to a volume of 25 mL or 10 mL to obtain the initial test concentrations (weight of HB/mL).

### 2.4. Total Phenolic Content (TPC) and Total Flavonoid Content (TFC) Analysis

The TPC was determined by the modified Folin–Ciocalteu method [13]. Briefly, 25.0 µL of each extract or gallic acid solution at different concentrations were mixed with 200.0 µL distilled water and 15.0 µL of Folin–Ciocalteu reagent, and allowed to react at room temperature for 3 min. Finally, 60.0 µL of aqueous solution of sodium carbonate (20.0%, *w*/*v*) was added, and the mixture was incubated in 96 well plates at room temperature for 30 min. The absorbance was read at 760 nm (Multiskan GO 1510, Thermo Fisher Scientific, Vantaa, Finland). The blank sample contained water instead of the extract or gallic acid solution. The total phenolic concentration was calculated from a calibration curve y = 0.0922x − 0.0199 (R^2^ = 0.9999), using gallic acid as a standard (0.52–8.33 µg/mL), and the obtained results were expressed as the gallic acid equivalents (GAE) (mg gallic acid per g of HB or plant material). The polyphenol content of the daily dose recommended by the manufacturer was also calculated. 

The TFC was determined according to the aluminum chloride colorimetric method, in which 100.0 µL of the extract or quercetin solution at different concentrations were mixed with 100.0 µL 2% methanolic solution of aluminum chloride (complexing reagent), and incubated in 96 well plates at room temperature for 10 min. The absorbance was read at 415 nm (Multiskan GO 1510, Thermo Fisher Scientific, Vantaa, Finland). The blank contained methanol instead of aluminum chloride solution. The TFC was calculated from a calibration curve y = 0.0262x − 0.0206 (R^2^ = 0.9998), using quercetin as a standard (1.625–50.0 × 10^−3^ mg/mL). The results were expressed as the quercetin equivalents (mg QE/g HB or plant material).

### 2.5. High-Performance Liquid Chromatography (HPLC) Analysis

Flavonoids and phenolic acids content was determined as described previously [14], using a Dionex HPLC system, with a PDA 100 detector, and a Hypersil Gold (C-18) column (5 μm, 250 × 4.6 mm, Thermo EC). The mobile phase consisted of 1% formic acid in water (A) and acetonitrile (B), in a gradient mode 5–60% B over 60 min. The compounds were identified by comparing their retention times and UV spectrum with the standards. Quantitative analysis was based on measuring the peak area regarding the appropriate standard curve prepared from five concentrations (0.0625–1 mg/mL). The results were expressed as the mg of compound/g HB or plant material.

### 2.6. Determination of Hypoglycemic Potential

#### 2.6.1. α-Glucosidase Inhibitory Assay

Inhibition of α-glucosidase by the extracts was performed according to Studzińska-Sroka et al. [15], with some modifications. Briefly, 50.0 μL of sample solution (5–40 mg/mL), acarbose (5–40 mg/mL), chlorogenic acid, gallic acid, protocatechuic acid and rutin as a positive control, (0.5–20 mg/mL), 50.0 μL of 0.1 M phosphate buffer (pH 6.8) and 30.0 µL of α-glucosidase solution (0.5 U/mL) were pre-incubated in 96 well plates at 37 °C for 15 min. Next, 20.0 μL of 5 mM p-nitrophenyl-α-D-glucopyranoside (pNPG) solution in 0.1 M phosphate buffer (pH 6.8) was added and incubated at 37 °C for 20 min. The reaction was terminated by adding 100.0 µL of sodium carbonate (0.2 M) into the mixture. The absorbance of the liberated p-nitrophenol was measured after 2 min at 405 nm. The absorbance of enzyme solution without extracts/acarbose served as the control, with total enzyme activity. The absorbance in the absence of the enzyme was used as the blind control. The absorbance of extract/compound solution without enzyme was used as the blank for tested sample. The enzyme inhibition rate (presented for the final concentration of substance in enzymatic reaction) was expressed as a percentage of inhibition and calculated using the following formula:

Inhibitory activity (%) = [(A_0_ − A_1_)/A_0_] × 100

where A_0_ is the absorbance of the control (100% enzyme activity), and A_1_ is the absorbance of the tested sample. 

#### 2.6.2. α-Amylase Inhibitory Assay

The α-amylase inhibitory activity of each herbal blends was determined by a spectrophotometric method. In the first step, 20 µL of α-amylase solution prepared by dissolving in phosphate buffer with pH = 6.9 (4.0 U/mL) and 20 µL of the test extract (80 mg/mL) or acarbose (0.008 mg/mL, used as a positive control) were preincubated in the 96-well plate at 37 °C. After 20 min, 20 µL of previously prepared in warm 0.1 M phosphate buffer (pH 6.9) and 0.5% starch solution was added to the wells and incubated again for 20 min at 37 °C. Then, 60 µL of color reagent was added to each well. Color reagent was containing 96 mM 3,5-dinitrosalicylic acid solution (20 mL), 5.31 M potassium sodium tartrate solution in 2 M sodium hydroxide (8 mL), and deionized water (12 mL). The plate was incubated at 85 °C for 15 min, then cooled to room temperature, and 80 µL of water was added. The measurement of absorbance was carried out at 540 nm (Multiskan GO 1510, Thermo Fisher Scientific, Vantaa, Finland). Absorbance of the enzyme solution without extracts/acarbose was used as a control for total enzyme activity. Individual blanks containing test herbal blends without the enzyme and starch solution were prepared for correcting the background absorbance. The rate of enzyme inhibition (presented for the final concentration of substance in enzymatic reaction) was expressed as a percentage of inhibition, and calculated using the following formula:

Inhibitory activity (%) = [(A_0_ – A_1_)/A_0_] × 100

where A_0_ is the absorbance of the control reduced by the sample background (100% enzyme activity), and A_1_ is the absorbance of the tested sample reduced by the sample background. 

### 2.7. Determination of the Preventive Potential for Type II Diabetes Complications

#### 2.7.1. DPPH and CUPRAC Assays 

The DPPH assay was effected according to [15] with slight modifications. Briefly, 25.0 μL of the extract at different concentrations were mixed with 175.0 μL of DPPH^•^ solution; then, the reaction mixture was shaken and incubated in the dark at room temperature for 30 min. DPPH^•^ solutions were freshly prepared for each analysis (3.9 mg DPPH in 50.0 mL of MeOH). Absorbance was measured at 517 nm. The control blank contained 25.0 μL of distilled water and 175.0 μL of DPPH^•^ solution. The inhibition of the DPPH^•^ radical by the sample was calculated according to the following formula:

DPPH scavenging activity (%) = [(A_0_ − A_1_)/A_0_] × 100%

where A_0_ is the absorbance of the control, and A_1_ is the absorbance of the sample. The IC_50_ values (expressed as final concentration in the sample), i.e., the amount of antioxidant necessary to half of the initial DPPH^•^ concentration, were used to compare the quality of the antioxidant potency of the studied extracts. A lower absorbance of the reaction mixture indicated a higher free radical scavenging activity.

The CUPRAC (cupric ion reducing antioxidant capacity) assay was effected according to Studzińska-Sroka et al. [15]. The stock solutions of CUPRAC reagent included equal parts of acetate buffer (pH 7.0), 7.5 mM neocuproine solution in 96% ethanol, and 10.0 mM CuCl_2_xH_2_O solution. Briefly, 50.0 μL of the extract at different concentrations was mixed with 150.0 μL of CUPRAC solution, then shaken and incubated at room temperature for 30 min in the dark condition. The absorbance was measured at 450 nm against blank sample (water mixed with CUPRAC solution). The results were expressed as the IC_0.5_, which corresponds to the concentration (expressed as final concentration in the sample) required to produce absorbance value equal 0.5. A higher absorbance of the reaction mixture indicated a higher antioxidant reducing capacity.

#### 2.7.2. Determination of Fe^2+^ Chelating Activity

The binding of Fe^2+^ by the extracts was effectuated according to Dinis et al. [16], with some modifications. Briefly, 200.0 μL of sample solution or EDTA (as a positive control), at different concentrations, and 10.0 μL of FeCl_2_·4H_2_O (1 mM) were mixed and pre-incubated in 96 well plates at room temperature for 10 min. Afterward, 10 μL of ferrozine (2.5 mM) was added and incubated at room temperature for 30 min. The absorbance of the iron (II)-ferrozine complex was measured at 562 nm. The chelating activity (presented for the final concentration of samples) was expressed as a percentage using the following equation:

Chelating activity (%) = [(A_0_ − A_1_)/A_0_] × 100%

where A_0_ is the absorbance of the control, and A_1_ is the absorbance of the sample. 

#### 2.7.3. Determination of Anti-Collagenase Potential

The collagenase inhibitory activity of each HBs was determined in vitro according to Widodo et al. [17], with some modifications. Briefly, 15.0 μL of enzyme, 60 μL of tricine buffer (pH 7.5), and 30 μL of HB extracts (10 mg/mL) or epigallocatechin gallate (EGCG) (1.0 mg/mL and 0.1 mg/mL, positive control) were mixed and incubated at 37 °C for 20 min. Next, 20 μL of FALGPA (0.5 mM) was added, and they were measured immediately at 325 nm using the plate reader (Multiskan GO 1510, Thermo Fisher Scientific, Vantaa, Finland), following the addition of the substrate, and then again after 20 min of incubation at 37 °C. The collagenase inhibition rate (presented for the final concentration of substance in enzymatic reaction) expressed as a percentage of inhibition was calculated using the following formula:

Inhibitory activity (%) = [(A_0_ – A_1_)/A_0_] × 100

where A_0_ is the absorbance of the control (100% enzyme activity), and A_1_ is the absorbance of the tested sample. 

### 2.8. Statistical Analysis

Analyses of determination of total flavonoids and phenolic compounds, as well as biological activity, were performed in six replicates; HPLC analysis was performed in three replicates. Results were expressed as means ± SD.

## 3. Results 

### 3.1. Phytochemical Characterization of Herbal Blends

The examined herbal tea blends have a diverse composition of plant ingredients (Table 1). Thus, they were characterized by diverse content of polyphenolic compounds. Our results showed that the examined herbal infusions are characterized by different total polyphenol and flavonoid contents (Table 2). The highest total polyphenol content, determined for HB5, HB1, and HB6 (16.06, 14.94, and 13.97 mg GAE/g HB, respectively), was correlated with a high content of flavonoids (2.23, 3.06, and 2.03 mg QE/g HB, respectively). Interestingly, although HB2 had also high total polyphenols content (15.30 mg GAE/g HB), no such correlation was noted in this case (Table 2). 

To complete the total compounds analysis results, we examined in the tested infusions some polyphenols’ contents (chlorogenic acid, gallic acid, protocatechuic acid, rutin), considered essential for antidiabetic activity [18]. The obtained results showed that the amount of selected compounds is varied in the investigated HBs (Table 3), with predomination of chlorogenic and gallic acids. Both protocatechuic acid and rutin were present in the samples in smaller amounts. 

### 3.2. Determination of Hypoglycemic Potential

α-glucosidase and α-amylase are some of the key enzymes involved in carbohydrate metabolism, which digest oligosaccharides (disaccharides and polysaccharides) or other complex carbohydrates into monosaccharides (glucose) [7]. By inhibiting the activity of these enzymes, the digestion of carbohydrates and the absorption of glucose in the small intestine are blocked. 

We evaluated the α-glucosidase inhibition potential of HBs at four different concentrations (1.7, 3.3, 6.7, and 13.3 mg/mL). The results indicate that the activity of the tested extracts was dose dependent. HB1, HB5, HB6, and HB7 showed the strongest inhibition of the enzyme (Table 4). It is worth noting that in the lowest tested concentration (1.7 mg/mL), only HB5 and HB7 inhibited the enzyme comparable to acarbose used at the same concentration (93.65%, 86.97%, and 80.83%, respectively) (Table 4). Additionally, the inhibitory activity of compounds detected in the tested HBs (chlorogenic acid, gallic acid, as well as protocatechuic acid, and rutin), was tested. For the most active compounds (chlorogenic acid and rutin), the tested concentrations were lower than those for the infusions (0.17 mg/mL and 0.3 mg/mL). However, gallic and protocatechuic acids acted much weaker, and only the concentration of 6.7 mg/mL allowed to observe their inhibitory effect on the tested enzyme. The results are presented in Table 4.

The α-amylase inhibition of HBs was performed at 27.6 mg/mL concentration. Our study demonstrated that the most potent inhibitor is HB7, and the weaker inhibitors are HB5, HB6, HB2, and HB1, respectively. HB3 and HB4 exhibit no inhibitory effect. However, the activity of tested HBs is significantly lower than that of acarbose. The results are presented in Table 5.

### 3.3. Determination of the Preventive Potential for Type II Diabetes Complications

#### 3.3.1. In Vitro Antioxidant Activity 

Oxidative stress is responsible for the development of diabetes complications. Therefore, antioxidant activity is of great importance in preventing adverse complications of this disease. To test the antioxidant activity of HBs’ extracts two complementary methods were chosen: the DPPH^•^ radical, characterizing the ability of the tested sample to scavenge free radicals, and the CUPRAC reagent, assessing the reducing properties of metal ions.

The results of the HBs infusions antioxidant activity analysis showed that among the tested preparations, the most active was HB1 (IC_50_ DPPH = 310.03 µg/mL, IC_0.5_ CUPRAC = 368.15 µg/mL) and HB5 (IC_50_ DPPH = 350.52 µg/mL, IC_0.5_ CUPRAC = 344.43 µg/mL) herbal infusions. However, the effect of both tested water extracts was weaker than that of vitamin C, used as a standard (IC_50_ DPPH = 7.62 µg/mL and IC_0.5_ CUPRAC = 14.64 µg/mL). HB4 (bean pod, nettle herb or leaf, nettle rhizome, dandelion root) performed much weaker than others, which indicates its poor antioxidant properties. The scavenging capacity of DPPH free radicals or the reducing properties of other tested preparations ranged from IC_50_ DPPH = 361.17 µg/mL to IC_50_ DPPH = 744.74 µg/mL, and from IC_0.5_ CUPRAC = 409.92 µg/mL to IC_0.5_ CUPRAC = 686.43 µg/mL (Table 6). The scavenging capacity of DPPH free radicals or the reducing properties of HB2 and HB3 was presented as approximately 2-times lower to the highest active herbal infusions (Table 6). The HB4 was characterised by the worst activity.

The relationship between the decompartmentalized metal ions and diabetic complications is the content of many scientific works [19]. To know the potential of herbal infusions in this area, we assessed the chelating properties of Fe^2+^ ions by HBs water extracts, using a ferrozine in vitro test. The obtained results indicate a dose-dependent, robust activity of the studied samples. The water extracts of HB3, HB4, and HB6 showed the most potent effect, showing > 50% of chelating activity at the lowest concentration tested (0.45 mg/mL), which was also 20 times lower than when taken in the form of an infusion. The chelating ability close to 90% was achieved by HB1-HB6 blends for the concentration four times lower (2.3 mg/mL) than the concentration of the ready-to-drink herbal infusion (Table 7). The only herbal blend that showed no activity (at low concentrations) or little activity at the highest concentrations tested was HB7 (Table 7). This result contrasted with HB7’s high anti-radical activity, as well as very good **α**-glucosidase and **α**-amylase inhibition properties and collagenase inhibition.

#### 3.3.2. In Vitro Anti-Inflammatory Activity

Our research has shown that the mixtures used to prepare infusions taken in the early stages of diabetes can inhibit collagenase, and the activity was recorded at a concentration that is intended for direct consumption. The HB5 mixture most strongly inhibited the enzyme (28.79%). The other blends, with the exception of HB3, which did not show any activity at the tested concentration, were less effective (8.47–21.46%). Despite the reported activity, the action level of the most active mixture was three times lower than the standard’s 10-fold lower concentration (EGCG) (Table 8).

### 3.4. Summary of Antidiabetic Potential of Tested Herbal Blends

**Figure 1:**
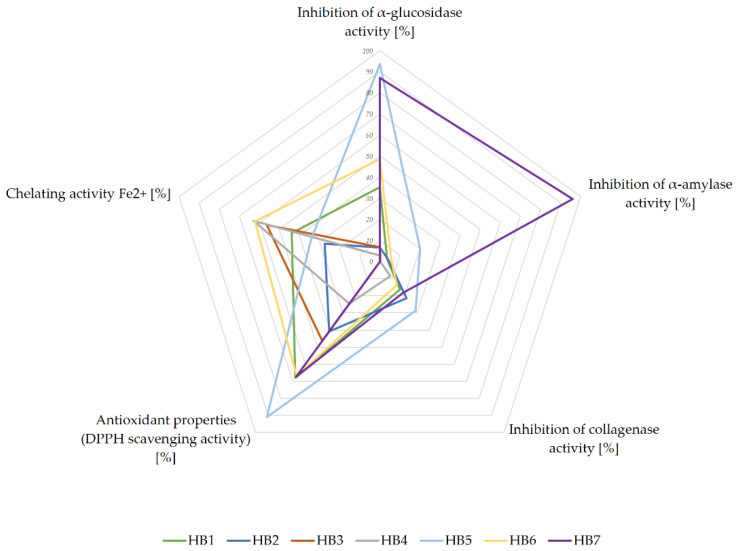
The antidiabetic potential of herbal blends 1–7 (HB 1–7), presented graphically, taking into account the measured biological properties expressed in %. The graph was made for the concentrations: inhibition of α-glucosidase activity 1.7 mg/mL; inhibition of α-amylase activity 26.7 mg/mL; inhibition of collagenase activity 2.4 mg/mL; antioxidant potential - DPPH assay 0.625 mg/mL; chelating activity 0.45 mg/mL.

Finally, to better visualize and compare the obtained results, we performed the data as a star diagram (Figure 1). According to the presented relationship, HB7 and HB5 have the highest antidiabetic potential. Both of them were characterized by high hypoglycemic properties, especially by inhibiting α-glucosidase, and also, but in a moderate way, α-amylase. HB5 is also characterized by preventive potential against diabetic complications, due to its highest antioxidant capacity and the best collagenase inhibition properties. Another HBs, whose biological properties (at least 2 out of 3 examined) suggest an interesting preventive potential, are HB6, HB1, and HB4, respectively, for which the chelating and antioxidant potential were noticeable.

## 4. Discussion

Diabetes is a metabolic disease that, if left untreated, leads to serious complications in the form of retinopathy, neuropathy, or nephropathy. Apart from the standard pharmacotherapy, the use of herbal drugs seems to be an interesting and complementary strategy to prevent or alleviate the symptoms of the disease. However, the effectiveness of multi-component HBs is often not scientifically proven and the mechanism of their activity also needs to be clarified. 

Our in vitro study evaluated the antidiabetic potential of herbal infusions to prevent and support the treatment of early stages of type II diabetes. The key question is whether the extraction with hot water is effective enough to receive biological activity of the active ingredients. The products selected for our study contained pharmaceutical raw materials (according to the European Pharmacopoeia: dandelion root, dandelion herb with root, couch grass rhizome, nettle leaf, cinnamon) [20], or the components of dietary supplements (chokeberry fruit, blackcurrant fruit, apple fruit, buckwheat husk, mulberry leaf, bean pod).

The antidiabetic mechanisms of polyphenols include the possibility of inhibiting digestive enzymes (e.g., α-amylase or α-glucosidase), which are essential in limiting the absorption of sugars [21]. Polyphenols have also been reported as stimulants of intestinal peptides secretion that can stimulate insulin secretion in the pancreas [22]. The data confirm the ability of polyphenolic compounds to stimulate insulin production from β-cells of the pancreas, increasing glycolysis and reducing gluconeogenesis, [23,24], scavenging free radicals, and chelating metals, which translates into anti-inflammatory activity [25].

The mentioned antidiabetic potential of polyphenolic compounds prompted us to conduct phytochemical characterization of the studied herbal infusions. HBs containing mulberry leaves (HB1, HB2, HB5, HB6) had high polyphenols and total flavonoids content. The highest TFC value was for single-component HB1 with *Mori albi folium* (mulberry leaves) (Table 2). Some literature data confirm that flavonoids are the important fraction of natural substances in the mulberry leaves, responsible for its biological activity [26]. 

More detailed analysis showed that all the tested herbal infusions contained chlorogenic acid (Table 3), the compound of high importance in terms of regulating blood glucose level [27] and alleviating metabolic syndrome symptoms [26,28] in a number of in vitro and in vivo studies. The therapeutic efficacy of chlorogenic acid, both as a pure substance and in the form of an extract was also proven in some clinical studies [29]. The highest content of chlorogenic acid was determined for HB1, with mulberry leaves as the only ingredient (Table 3). Six out of the seven studied HBs contained gallic acid, whose antidiabetic potential has been supported by various studies [30,31]. The highest content of this phenolic compound was observed in the herbal infusions containing mulberry leaves (HB1, HB5, HB6), which is consistent with previous literature data [32]. Protocatechuic acid and rutin were presented in the tested HBs in small amounts (Table 3). The low concentration of rutin is due to the tested extracts’ water nature, and the low solubility of the flavonoid in water [33,34].

The inhibition of α-glucosidase is a known mechanism of hypoglycemic activity. The chemical glucosidase inhibitors available on the pharmaceutical market are characterized by many side effects associated with gastrointestinal discomforts, such as abdominal distention, vomiting, and diarrhea [35], while some α-glucosidase inhibitors of plant origin can show hypoglycemic activity without the appearance of such side effects [36]. Therefore, we tested herbal infusions from HBs for their ability to inhibit α-glucosidase and α-amylase. Our study showed the α-glucosidase and α-amylase inhibitory potential of HBs. We noticed these properties are probably connected with the high quantity in all tested herbal infusions of phenolic compounds (total polyphenols and flavonoids, as well as individual phenolic acids: chlorogenic acid, gallic acid, protocatechuic acid, and rutin). We demonstrated significant inhibitory activity of examined pure substances for α-glucosidase, which is consistent with the results of other authors [18]. According to literature data, the active compounds we detected in the herbal infusions exhibit an inhibitory effect on α-amylase activity [37,38]. 

Subsequently we noticed that the potency of tested HBs in α-glucosidase and α-amylase inhibition is stronger with the increased amount of cinnamon in bark their composition, which was especially observed for HB5 and HB6 (Table 4 and Table 5). We observed that HB5 containing 30% of cinnamon bark showed 98.06% of α-glucosidase inhibition at the concentration 3.3 mg/mL, while 5% of the bark in HB6 presented 73.71% of inhibitory activity at the same concentration. A similar effect was noticed for α-amylase inhibition, with HB5 showing 20.14% of enzyme inhibition in concentration 26.7 mg/mL, while HB6 presented 5.62% of enzyme inhibition in concentration 8.0 mg/mL. The strong ability to inhibit α-glucosidase and α-amylase by cinnamon bark has been described by other authors [39,40]. Moreover, an additive effect on the inhibition of intestinal enzymes (α-glucosidase and α-amylase) was demonstrated for cinnamon bark combined with acarbose [40]. The cinnamon bark phenolic acids’ synergism with metformin was also observed [41]. The studies shown that the antidiabetic potential of cinnamon bark was highly correlated with their proanthocyanidin and condensed tannin [42]. Moreover the presence of flavonoids in mulberry leaves as the constituent of HB5 and HB6 water herbal infusions favors inhibiting the enzyme activity [43]. However, this issue needs further studies to explain the mutual relationships between the components of the herbal blends and the observed activity.

The HB7, containing anthocyanins-rich plants in, indicated high potential to inhibit the α-glucosidase and α-amylase enzymes. Sui et al. [44] demonstrated in vitro inhibitory effects of anthocyanins against pancreatic α-amylase verified, also by an in silico molecular docking study. Barik et al. [45] suggest anthocyanins regulate postprandial hyperglycemia not solely by inhibiting α-glucosidase, but also as a result of modulating glucose uptake and sugar transporters. Moreover, Boath et al. [46] show that the blackcurrant, constituting the important part of HB7, has strong ability to inhibit α-glucosidase. What is more, it synergistic effect with acarbose was also observed [46]. It is also known that both chokeberry and blackcurrant fruit contain active flavonoids and tannins, which affect the antidiabetic potential by different mechanisms, including inhibition of the enzymes [12].

Cells’ oxidative stress contributes to the development of insulin resistance and also causes changes in the walls of blood vessels, which, in the conditions of increased hyperglycemia, leads to complications of diabetes [47]. Conducted experiments show that herbal infusions can exhibit antioxidant properties that can prevent diabetes complications and protect, among others, before the oxidation of unsaturated lipids and promoting oxidative damage at different levels.

In our study, high antioxidant activity (however, weaker than vitamin C used as a standard) was noted for most of the herbal infusions prepared from tested HBs (Table 6). What is more, these results correlated with the high total polyphenols or total flavonoid contents (Table 2), as well as with high content of chlorogenic and gallic acid, compared to other tested infusions (Table 3). Moreover, the anthocyanins and condensed proanthocyanidins, compounds of blackcurrant or chokeberry fruits [48] (the components of HB7), could explain the antioxidant potential of HB7. The HB5 and HB6, in addition to mulberry leaves, also contained cinnamon bark, a valuable source of antioxidant and hypoglycemic compounds [49]. Our research indicated no relationship between the increased content of cinnamon bark in the two-component blends (mulberry leaf and cinnamon bark) and their antioxidant properties.

The ability to chelate metal ions increases total antioxidant potential [50] which may result in a reduction in the concentration of the catalyzing transition metal in lipid peroxidation. This approach determined the emergence of therapies in which the use of substances can chelate transition metals aided in the treatment of chronic hyperglycemia [51]. The chelating abilities of metals are mainly due to polyphenol compounds [52], including chlorogenic, gallic, or protocatechuic acids and flavonoids [42,52,53]. For HB1, HB5, and HB6, the dominant component was the polyphenol-rich mulberry leaves, which can influence high chelating abilities. The chelating properties of cinnamon bark (a component of HB5 and HB6) have been proven for cinnamaldehyde present in the raw material in large amounts [54]. Our research shows that quite possibly not only polyphenols have an impact on the assessed activity. The composition of the chelating active HB3 and HB4 blends suggests that the large amounts of *Phaseoli pericarpium* (among others sources of amino acids, chromium salts, guanidine derivatives, triterpenes, condensed tannins, sterols, and small quantities of polyphenols) [55,56] and aerial parts from *Urtica dioica* (besides polyphenols, the source of amines, amino acids, terpenes, organic acids, sterols) [57,58] can affect the increase of the ability of chelation. Carrasco-Castilla et al. [59] state that peptide fractions isolated from *Phaseolus vulgaris* showed the ability to chelate iron ions in vitro. According to Drużyńska et al. [56], *Phaseoli pericarpium*’s condensed tannins increase the chelation of transition metal ions. The high capacity of aqueous extracts from aerial parts of *Urtica dioica* to chelate iron ions was demonstrated by Gülçin et al. [60].

The results of the analysis concerning HB7, active in other trials, but with the weakest chelating ability among the tested HBs, seem interesting. This may be explained by the substantial amounts of dried chokeberry and blackcurrant fruits, rich in anthocyanins, the reduced chelating ability of which is related to the presence of one- or two-metal binding sites, when compared the other polyphenols, with three or four metal-binding sites [52]. 

Collagenase, belonging to the group of metalloproteinases, is an enzyme whose activity in tissues increases significantly in diabetic disease [61,62], that results in the development of complications related to the disturbance of collagen metabolism in periodontal diseases [61], vascular complications [63,64,65], or inflammation of the gastrointestinal tract [62]. The compounds of plant origin, both as pure substances and in the form of plant extracts, can effectively inhibit metalloproteinases [66,67], which was especially proven for flavonoids (including rutin and potent inhibitor of collagenase EGCG [68]), phenolic acids (in chlorogenic acid and gallic acid), anthocyanins (including from blackcurrant (delphinidin derivatives)) or procyanidins [69]. Moreover, collagenase-inhibiting properties were noted for extracts from nettle [70], and black chokeberries [71], present in the examined HBs. Our results indicated moderate anti-collagenase activity of all the tested HBs, with the exception of HB3. Therefore, it seems that the regular consumption of herbal infusions may help in the likely consequences of hyperglycemia, such as inflammation of the oral cavity or further digestive tract sections. Moreover, it should be noted that the absorption capacity of the active substances present in HBs also provides the possibility of systemic action, which increases the effectiveness of the prevention of diabetic complications. However, this requires further research.

## 5. Conclusions

We have proven that the extraction with hot water during the preparation of herbal infusions allows for the achievement of adequate concentrations of phenolic acids, flavonoids, and other probably synergistic compounds to provide the potential for hypoglycemic action. The examined herbal blends demonstrated a multidirectional mechanism of action, and are helpful in the prevention or treatment of early stages not only of diabetes, but also other metabolic diseases leading to several complications. With the obtained results, we can recommend herbal infusions containing mulberry leaves, especially those containing cinnamon bark or anthocyanins, to support the treatment of diabetes type II and to prevent its grave implications.

## Figures and Tables

**Table 1:** Composition of the herbal tea blends.

Herbal Blend	Herbals Content of the Preparations
HB1	*Mori albi folium* 100%
HB2	*Mori albi folium* 25%, *Phaseoli pericarpium* 25%, *Fagopyrum esculentum squama* 25%, *Taraxaci radix* 12.5%, *Urticae folium* 12.5%
HB3	*Phaseoli pericarpium* 40%, *Urticae herba* 17%, *Mori albi folium* 15%, *Taraxaci herba* 15%, *Graminis rhizoma* 13%
HB4	*Phaseoli pericarpium* 40%, *Urticae herba vel Urticae folium* 30%, *Graminis rhizoma* 20%, *Taraxaci herba et radix* 10%
HB5	*Mori albi folium* 70%, *Cinnamomi cortex* 30%
HB6	*Mori albi folium* 95%, *Cinnamomi cortex* 5%
HB7	*Ribes nigrum fructus* 29%, *Aronia fructus* 29%, *Mali fructus* 26%, *Mori albi folium* 15,4%, *Fagopyrum esculentum squama* 0.3%, *Phaseoli pericarpium* 0.3%

**Table 2:** Content of polyphenols (TPC) and flavonoids (TFC) and their daily dose in the tested extracts from herbal tea blends.

Herbal Blend	Content of Active Substances		Content of Active Substances in the Daily Dose Recommended by the Manufacturer	
	TPC (mg GAE/g HB)	TFC (mg QE/g HB)	Polyphenols (mg/day)	Flavonoids (mg/day)
HB1	14.94 ± 0.24	3.06 ± 0.05	89.64	18.36
HB2	15.30 ± 0.30	1.30 ± 0.06	61.20	5.20
HB3	11.89 ± 0.36	1.43 ± 0.05	35.67	4.29
HB4	3.83 ± 0.17	0.56 ± 0.06	30.64	4.48
HB5	16.06 ± 0.63	2.23 ± 0.03	96.36	13.38
HB6	13.97 ± 0.53	2.03 ± 0.03	83.82	12.18
HB7	10.92 ± 0.42	1.08 ± 0.07	98.28	9.72

HB: herbal blend; GAE: gallic acid equivalents; QE: quercetin equivalents.

**Table 3:** Content of selected polyphenolic compounds in herbal blends.

Herbal Blend	Content of Selected Polyphenolic Compounds (mg/g Herbal Blend)			
	Chlorogenic Acid	Gallic Acid	Protocatechuic Acid	Rutin
HB1	1.490 ± 0.057	0.275 ± 0.009	0.060 ± 0.010	0.236 ± 0.015
HB2	0.474 ± 0.026	0.079 ± 0.007	0.040 ± 0.010	0.071 ± 0.007
HB3	0.722 ± 0.040	0.046 ± 0.003	n.a.	0.058 ± 0.010
HB4	0.079 ± 0.009	n.a.	0.070 ± 0.010	0.043 ± 0.006
HB5	0.407 ± 0.028	0.462 ± 0.014	n.a.	0.029 ± 0.008
HB6	0.107 ± 0.015	0.363 ± 0.021	0.147 ± 0.015	n.a.
HB7	1.896 ± 0.143	0.066 ± 0.009	n.a.	0.100 ± 0.023

n.a.: not active.

**Table 4:** Inhibition of α-glucosidase activity by the infusions from herbal blends.

**Herbal Blend**	**Inhibition of α-Glucosidase Activity [%]**			
	**Concentration**			
	**1.7 mg/mL**	**3.3 mg/mL**	**6.7 mg/mL**	**13.3 mg/mL**
HB1	35.26 ± 1.11	83.02 ± 1.72	92.82 ± 0.52	98.84 ± 1.48
HB2	6.73 ± 1.23	12.43 ± 2.28	44.60 ± 1.03	68.49 ± 4.69
HB3	6.91 ± 0.80	17.86 ± 5.81	29.70 ± 1.12	57.30 ± 5.55
HB4	2.87 ± 0.95	8.83 ± 0.50	17.13 ± 2.00	37.38 ± 3.59
HB5	93.65 ± 1.11	98.06 ± 0.90	99.78 ± 0.24	100.00
HB6	48.66 ± 3.51	73.71 ± 0.55	81.81 ± 1.78	88.14 ± 1.14
HB7	86.97 ± 5.63	97.27 ± 0.37	99.82 ± 0.26	99.22 ± 0.94
Acarbose	80.83 ± 1.02	88.07 ± 0.77	91.98 ± 0.17	93.73 ± 0.03
**Active Compound**	**Concentration**			
	**0.17 mg/mL**	**0.33 mg/mL**	**3.3 mg/mL**	**6.7 mg/mL**
Chlorogenic acid	46.44 ± 1.02	81.35 ± 3.34	n.d.	n.d.
Gallic acid	n.d.	n.d.	18.00 ± 5.09	99.65 ± 2.34
Protocatechuic acid	n.d.	n.d.	0.46 ± 1.42	99.99 ± 0.13
Rutin	65.33 ± 3.42	97.07 ± 0.55	n.d.	n.d.

n.d.: not determined.

**Table 5:** Inhibition of α-amylase activity by the infusions from herbal blends.

Herbal Blend	Inhibition of α-Amylase Activity [%]	
	Concentration	
	26.7 mg/mL	0.00267 mg/mL
HB1	3.62 ± 4.95	n.d.
HB2	4.02 ± 3.96	n.d.
HB3	0.31 ± 0.77	n.d.
HB4	0.13 ± 0.31	n.d.
HB5	20.14 ± 5.94	n.d.
HB6	5.62 ± 7.32	n.d.
HB7	96.16 ± 6.00	n.d.
Acarbose	n.d.	28.11 ± 2.80

n.d.: not determined.

**Table 6:** Antioxidant activity of tested herbal infusions.

Herbal Blend	Antioxidant Activity	
	DPPH^•^ (IC_50_ µg/mL) ^1^	CUPRAC (IC_0.5_ µg/mL) ^1^
HB1	310.03	368.15
HB2	715.58	686.43
HB3	744.74	680.14
HB4	1336.03	931.00
HB5	350.52	344.43
HB6	394.76	409.92
HB7	361.17	591.50
vitamin C	7.62	14.64

^1^ Concentration was expressed as mg of herbal blend in the tested sample.

**Table 7:** Chelating activity of tested herbal infusions.

Herbal Blend	Chelating Activity Fe^2+^ [%]				
	Concentration				
	0.45 mg/mL	0.9 mg/mL	2.3 mg/mL	4.6 mg/mL	9.1 mg/mL
HB1	43.92 ± 1.34	70.36 ± 7.02	92.01 ± 0.68	95.55 ± 0.63	99.34 ± 1.38
HB2	27.53 ± 3.05	51.16 ± 1.80	89.49 ± 2.45	89.85 ± 1.75	87.98 ± 0.10
HB3	56.38 ± 5.93	89.47 ± 1.74	96.58 ± 0.79	96.15 ± 1.23	97.11 ± 0.57
HB4	62.81 ± 1.72	88.31 ± 0.35	94.05 ± 0.48	94.35 ± 0.28	93.21 ± 0.53
HB5	34.15 ± 1.14	67.49 ± 1.90	94.11 ± 0.58	94.65 ± 0.42	97.15 ± 1.64
HB6	62.10 ± 3.16	91.10 ± 1.17	98.38 ± 0.54	98.41 ± 1.06	99.44 ± 2.99
HB7	n.a.	n.a.	5.91 ± 4.33	9.43 ± 4.72	9.33 ± 3.19
Reference	Concentration				
	0.01 mg/mL	0.023 mg/mL	0.045 mg/mL	-	-
EDTA	66.50 ± 2.20	99.59 ± 0.57	100.24 ± 0.20	-	-

n.a.: not active.

**Table 8:** Inhibition of collagenase activity by the infusions from herbal blends.

Herbal Blend	Inhibition of Collagenase Activity [%]		
	Concentration		
	2.4 mg/mL	0.24 mg/mL	0.024 mg/mL
HB1	16.22 ± 3.22	n.d.	n.d.
HB2	21.46 ± 3.04	n.d.	n.d.
HB3	n.a.	n.d.	n.d.
HB4	8.47 ± 4.66	n.d.	n.d.
HB5	28.79 ± 2.65	n.d.	n.d.
HB6	13.68 ± 6.21	n.d.	n.d.
HB7	18.42 ± 7.68	n.d.	n.d.
EGCG	n.d.	84.30 ± 8.83	35.78 ± 5.52

n.a.: not active; n.d.: not determined; EGCG: epigallocatechin gallate.

## Data Availability

The data supporting reported results were be found in: Department of Pharmacognosy, Poznan University of Medical Sciences and Department of Pharmacognosy, Faculty of Pharmacy, Jagiellonian University Medical College.
